# Supplementary material for: Asynchronous magnetic resonance elastography: Shear wave speed reconstruction using noise correlation of incoherent waves
Source: Magn Reson Med. 2022 Oct 27;89(3):990–1001. doi: 10.1002/mrm.29502 (PMC9792433; doi:10.1002/mrm.29502)
Supplement: Supplementary file 1 — DATA S1 MATLAB code used to generate the numerical simulations of Figure 1 [file MRM-89-990-s002.zip › k-Wave/helpfiles/example_us_bmode_phased_array.html]

Simulating B-mode Images Using A Phased Array Example (k-Wave)


# Simulating B-mode Images Using A Phased Array Example

This example illustrates how k-Wave can be used for the simulation of B-mode ultrasound images using a phased-array or sector transducer. It builds on the Simulating B-mode Ultrasound Images Example.

- Open the file in the MATLAB Editor
- Run the file in MATLAB

To allow the simulated scan line data to be processed multiple times with different settings, the simulated RF data is saved to disk. This can be reloaded by setting `run_simulation = false` within the example m-file. The data can also be downloaded from http://www.k-wave.org/datasets/example\_us\_phased\_array\_scan\_lines.mat

## Contents

- Running the simulation
- Scan conversion

## Running the simulation

The simulation is run in a similar same way to Simulating B-mode Ultrasound Images. In this example, a 64 element sector transducer is defined with all the elements active. This is used to replace both the `source` and `sensor` inputs. The scan lines are then simulated sequentially, with the value for `transducer.steering_angle` modified for each scan line.

```
% loop through the range of angles to test
for angle_index = 1:number_scan_lines

    % update the current steering angle
    transducer.steering_angle = steering_angles(angle_index);

    % run the simulation
    sensor_data = kspaceFirstOrder3D(kgrid, medium, transducer, transducer, input_args{:});

    % extract the scan line from the sensor data
    scan_lines(angle_index, :) = transducer.scan_line(sensor_data);

end
```

## Scan conversion

After simulation, the scan lines are processed before display following the steps described in Simulating B-mode Ultrasound Images. However, in this example, the scan lines are taken at different steering angles. This means the scan lines must be converted from polar coordinates to Cartesian coordinates. This step is performed using `scanConversion`

```
% set the desired size of the image
image_size = [Nx * dx, Ny * dy];

% convert the data from polar coordinates to Cartesian coordinates for
% display
b_mode_fund = scanConversion(scan_lines_fund, steering_angles, image_size, c0, kgrid.dt);
b_mode_harm = scanConversion(scan_lines_harm, steering_angles, image_size, c0, kgrid.dt);
```

The scattering phantom along with the simulated ultrasound images are shown below.
